# Human OTOP3 requires strong acidification for activation and exhibits mixed proton-anion permeation

**DOI:** 10.1016/j.jbc.2026.113454

**Published:** 2026-08-14

**Authors:** Tiago D.C. Morais, Fatemeh M. Badizi, Itai Itzhak, Moushumi A. Mou, Justin Chan, Anonnya Debi, Zhifei Wang, Yong Yu

**Affiliations:** Department of Biological Sciences, St John’s University, Queens, New York, USA

**Keywords:** OTOPetrin, OTOP, ion channel, proton channel, selectivity, gating, proton, chloride, electrophysiology, *Xenopus* oocytes

## Abstract

The OTOPetrin (OTOP) proton channel family comprises three members: OTOP1, OTOP2, and OTOP3. While OTOP1 has been established as the primary sour taste receptor, the physiological roles of OTOP2 and OTOP3 remain largely unknown. Although OTOP channels are widely recognized as proton-selective ion channels, their functional diversity is not fully understood. Here, we show that human OTOP3 (hOTOP3) exhibits distinct gating and permeation properties compared with mouse OTOP3 (mOTOP3) and other OTOP family members. Using two-electrode voltage clamp recordings in *Xenopus* oocytes, we found that hOTOP3 requires stronger extracellular acidification for activation, indicating a higher threshold for proton-dependent gating. We further identify the extracellular loop between the fifth and the sixth transmembrane segments (L5-6) contributes to this difference in pH sensitivity. Notably, under strongly acidic conditions, hOTOP3 exhibits a measurable anion permeability, a property not observed in human or mouse OTOP1 or OTOP2, nor in mOTOP3. Mutational analysis reveals coordinated effects on proton and anion currents, suggesting that the two permeation processes are mechanistically coupled. Together, these findings demonstrate that hOTOP3 exhibits a higher activation threshold and condition-dependent mixed proton-anion permeation. These results expand the functional diversity of the OTOP channel family and suggest that hOTOP3 may be adapted to function in highly acidic microenvironments, where coupled proton and anion flux could help maintain electrochemical homeostasis.

Proton permeability across cell membranes plays a critical role in regulating intracellular and extracellular pH, membrane potential, and many other physiological processes, including epithelial transport and sensory signaling (1, 2). Unlike other ions, protons are conducted through a limited number of specialized proton channels that exhibit high selectivity (2, 3). Understanding how proton channels achieve both controlled activation and selective permeation remains a fundamental question in membrane biology.

The OTOPetrin (OTOP) family has recently been identified as a new class of proton-selective ion channels (4). The three members of this family, OTOP1, OTOP2, and OTOP3, exhibit distinct expression patterns and physiological roles (4, 5). OTOP1 is expressed in vestibular and sour-sensing taste receptor cells and in multiple peripheral tissues, whereas OTOP2 is most abundant in the stomach, testis, and olfactory bulb, and OTOP3 expression is detected in the epidermis, small intestine, stomach, and retina (4, 6, 7). OTOP1 functions as the primary receptor for sour taste in the taste receptor cells and plays a key role in otoconia development in the vestibular system (4, 8, 9, 10). Compared to OTOP1, the physiological functions of OTOP2 and OTOP3 remain largely unknown. Nevertheless, structural and functional studies have shown that all three OTOP channels are activated by protons and exhibit strong proton selectivity (4, 11, 12, 13, 14). These findings have led to the prevailing view that these channels function as dedicated proton conductors. Unlike voltage-gated proton channels such as Hv1, which are activated by membrane depolarization and pH gradients (2, 3), OTOP channels are activated directly by extracellular acidification (4, 13, 14), suggesting distinct mechanisms of gating and physiological function.

Despite these advances, the functional diversity within the OTOP family remains incompletely understood. Given the sequence divergence among OTOP family members and their distinct expression patterns, it is possible that different OTOP channels are tuned to operate under different physiological conditions. More specifically, it remains unknown whether ion selectivity in OTOP channels is strictly fixed or can change in a state-dependent manner during channel activation. Studies in other ion channel families have demonstrated that channel gating can modulate ion permeation and, in some cases, alter apparent ion selectivity in a state-dependent manner (15, 16, 17), raising the possibility that similar mechanisms may operate in OTOP channels.

Previous reports have shown that OTOP3 exhibits distinct proton-gating properties and Zn^2+^ regulation compared to OTOP1 and OTOP2 (13, 18). In this study, we sought to define the gating and permeation properties of human OTOP3 (hOTOP3) and to compare them with those of mouse OTOP3 (mOTOP3) and other OTOP family members. Using two-electrode voltage clamp recordings in *Xenopus laevis* oocytes, we examined the pH dependence of channel activation, ion selectivity, and the effects of targeted mutations. We further employed domain-swapping approaches to identify structural determinants underlying species-dependent differences in gating. Our results reveal that hOTOP3 exhibits distinct properties in both proton-dependent gating and ion selectivity compared with mOTOP3 and other OTOP channels. These findings establish hOTOP3 as a functionally specialized member of the Otop family and suggest that hOTOP3 may be adapted to function in highly acidic physiological environments.

## Results

### Human OTOP3 requires strong acidification for activation compared with mouse OTOP3

**Figure 1:**
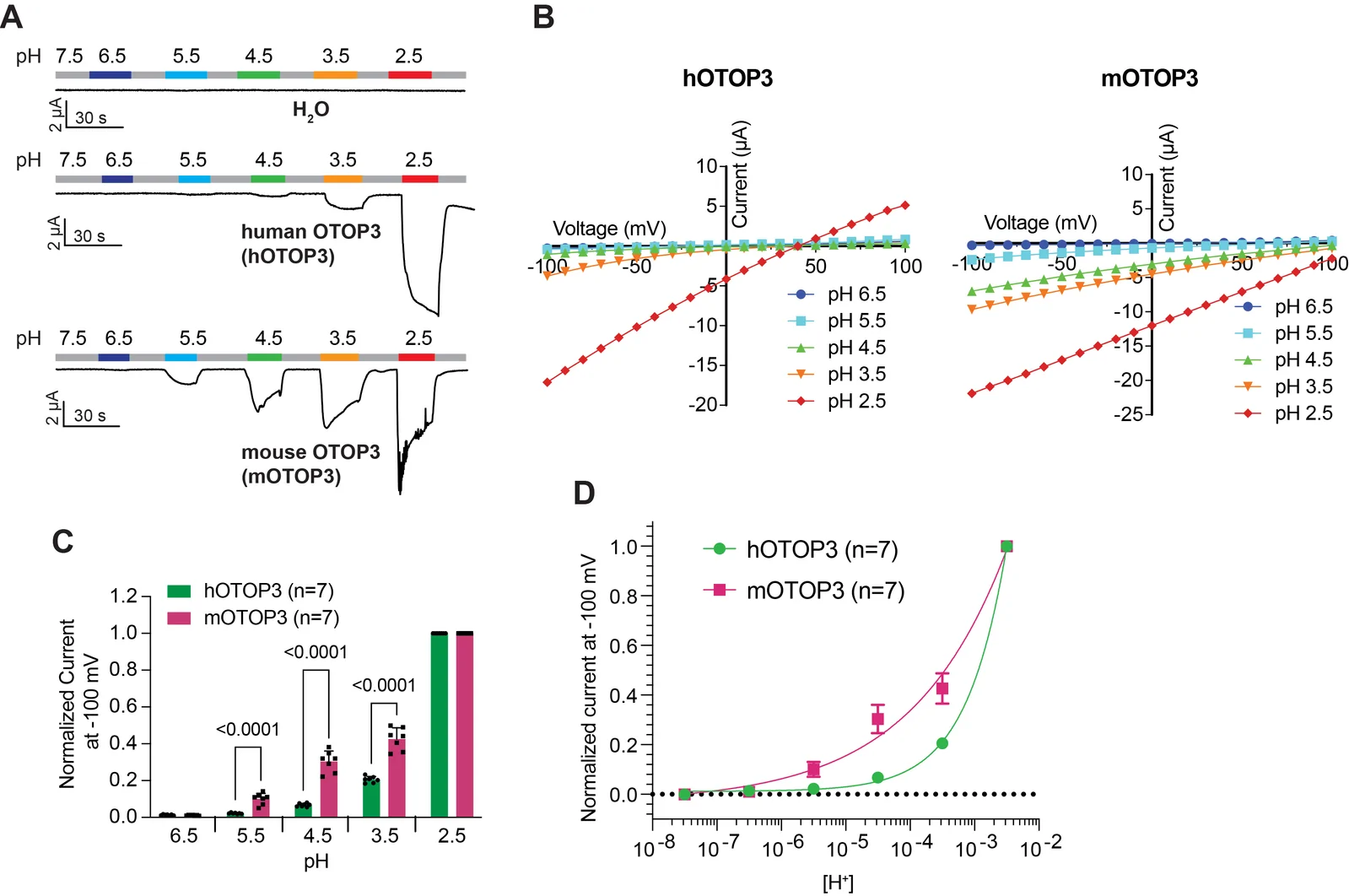
**Human OTOP3 requires stronger extracellular acidification for activation than mouse OTOP3.***A*, representative two-electrode voltage clamp recordings from *Xenopus* oocytes injected with water, human OTOP3 (hOTOP3), or mouse OTOP3 (mOTOP3). Oocytes were held at −60 mV and sequentially perfused with solutions of decreasing extracellular pH as indicated. Scale bars: 2 μA, 30 s. *B*, representative current–voltage (I-V) relationships of hOTOP3 (*left*) and mOTOP3 (*right*) measured at the indicated extracellular pH values. *C*, bar graphs show the quantification of normalized current amplitudes measured at −100 mV (mean ± SD, n = 7). Currents were normalized to the current recorded at pH 2.5 for each oocyte. Statistical significance was assessed using two-way ANOVA with Sidak’s multiple-comparisons test. Statistically significant comparisons are indicated by their corresponding *p* values; comparisons without labels were not statistically significant. Each data point was obtained from individual oocyte in this and the following figures. *D*, proton concentration–response relationships derived from normalized currents at −100 mV for human and mouse OTOP3 channels (mean ± SD).

In our preliminary studies, we found that hOTOP3 produced little to no detectable current within the pH range typically used to activate other OTOP channels (pH ≥ 4.0) (4, 13, 14, 18, 19). To further characterize its properties, we examined its activation under more strongly acidic conditions and directly compared its proton sensitivity with that of mOTOP3. To address this, hOTOP3 and mOTOP3 were expressed in *Xenopus laevis* oocytes, and currents were measured using two-electrode voltage clamp (TEVC) while holding the oocytes’ membrane potential at −60 mV and sequentially lowering pH from 7.5 to 2.5 in a bath solution containing 100 mM N-Methyl-D-glucamine chloride (NMDG-Cl) (Fig. 1*A*).

While water-injected oocytes showed no detectable currents under these conditions, in oocytes expressing OTOP3 channels, extracellular acidification induced inward currents that increased as the pH was lowered. In our testing, we noticed clear differences in activation between the two orthologs (Fig. 1*A*). In oocytes expressing mOTOP3, inward proton currents were first detected at pH 5.5 and became prominent at pH 4.5, consistent with previously reported acid responses of mOTOP3 and other OTOP channels (4, 11, 13, 14). As expected, further lowering the extracellular pH to 3.5 and 2.5 led to a progressive increase in inward current (Fig. 1*A*). In contrast, hOTOP3 produced no detectable current at pH 5.5 and only minimal currents at pH 4.5, requiring more pronounced acidification (pH 3.5 and 2.5) to elicit robust currents (Fig. 1*A*). Notably, once activated at pH 2.5, hOTOP3 generated currents that were comparable in magnitude to those of mOTOP3, suggesting that the lack of activation at moderate pH is unlikely due to reduced channel expression or intrinsic conductance but rather reflects a shift in proton-dependent gating.

Current–voltage relationships (I-V curves) measured at different extracellular pH values further illustrate this difference (Fig. 1*B*). In these experiments, oocytes were clamped at +50 mV, and currents were recorded using a voltage-step protocol from +100 mV to −100 mV to minimize proton efflux at positive voltages arising from intracellular proton accumulation, which can otherwise distort reversal potential measurements. For both channels, currents increased as extracellular pH decreased. However, at intermediate pH values (pH 5.5, 4.5, and 3.5), mOTOP3 exhibited larger inward currents than hOTOP3 (Fig. 1*B*). We also noted that the reversal potentials (V_rev_) of mOTOP3 currents were strongly positive, a feature characteristic of OTOP channels reflecting their high proton selectivity (4, 12, 14). In contrast, hOTOP3 produced clear currents only at pH 3.5 and 2.5, with almost no detectable activity at higher pH values. Additionally, at pH 2.5, the reversal potentials of hOTOP3 currents were relatively negative compared to those of mOTOP3, suggesting reduced proton selectivity (Fig. 1*B*). This feature is examined further later in the study.

To quantify these differences, the currents recorded at −100 mV under different pH conditions were normalized to the current elicited at pH 2.5 in each oocyte (Fig. 1*C*). At pH 5.5, 4.5, and 3.5, normalized currents of mOTOP3 were significantly larger than those of hOTOP3. Plotting normalized current as a function of proton concentration revealed a rightward shift of the activation curve for hOTOP3 relative to mOTOP3 (Fig. 1*D*), indicating that human OTOP3 requires a higher proton concentration (lower pH) for activation.

Collectively, these findings show that hOTOP3 requires substantially higher proton concentrations—roughly 10 times higher (corresponding to a 1-unit shift in pH)—for activation than mOTOP3.

### The S5–S6 loop contributes to the proton sensitivity of OTOP3 channels

**Figure 2:**
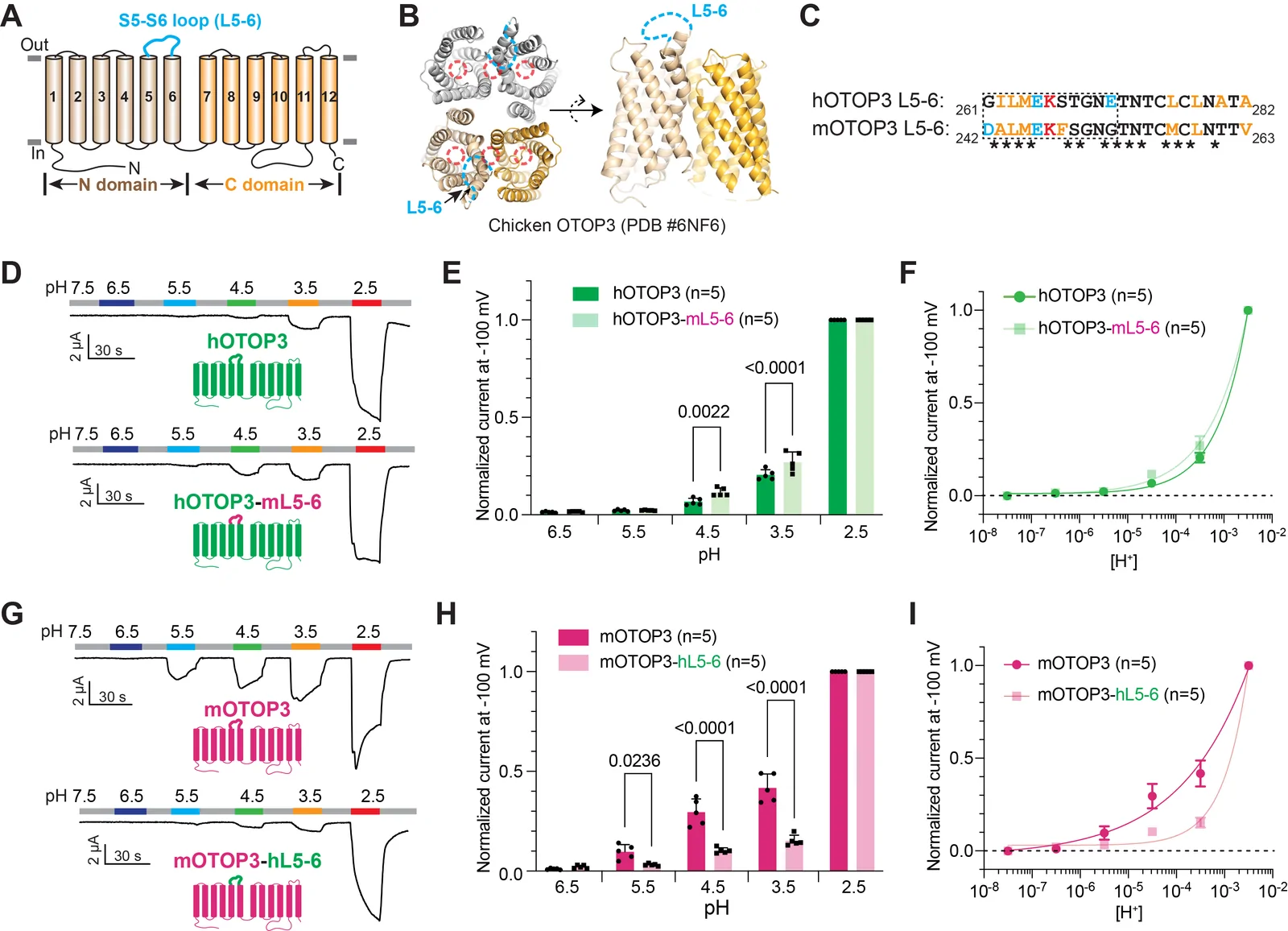
**The S5–S6 loop contributes to species-dependent difference in activation between human and mouse OTOP3.***A*, Schematic topology of OTOP channels showing 12 transmembrane segments (S1-S12) organized into N and C domains. The extracellular S5–S6 loop (L5-6) is indicated. *B*, *top* (*left*) and side (*right*) views of the cryo-EM structure of chicken OTOP3 (PDB: 6NF6 (11)). The L5-6 was not resolved in the cryo-EM structure, and its position is indicated with a dashed line in blue. Three possible proton-conducting pores in each monomer are indicated with dashed circles in *red*. *C*, sequence alignment of L5-6 from human and mouse OTOP3. Asterisks indicate identical residues. Hydrophobic, positively charged, and negatively charged residues are colored *orange*, *red*, and *blue*, respectively. The dashed box marks the region exchanged in the chimeric constructs. *D and G*, representative TEVC recordings from *Xenopus* oocytes expressing wild-type OTOP3 or chimeric channels in which the L5-6 was swapped between human and mouse OTOP3. Oocytes were sequentially perfused with solutions of decreasing extracellular pH as indicated. Scale bars, 2 μA and 30 s. *E, H*, quantification of normalized currents measured at −100 mV for wild-type and chimeric channels (mean ± SD; sample numbers are indicated in parentheses). Currents were normalized to the current recorded at pH 2.5 for each oocyte. Statistical significance was assessed using two-way ANOVA with Sidak’s multiple-comparisons test. Statistically significant comparisons are indicated by their corresponding *p* values; comparisons without labels were not statistically significant. *F, I*, Proton concentration–response relationships derived from normalized currents at −100 mV for the indicated channels (mean ± S*D*).

The overall topology of OTOP proteins consists of twelve transmembrane segments. Published cryo-EM structures of OTOP channels show that they form dimers, with each monomer comprising N-terminal (S1–S6) and C-terminal (S7–S12) domains that assemble into similar barrel-like structures (11, 12, 19) (Fig. 2*A*). Three possible proton conduction pathways have been proposed: one within the N barrel, one within the C barrel, and one located at the intrasubunit interface between the N and the C barrels (11, 12) (Fig. 2*B*).

Previous structural and functional studies of OTOP channels have suggested that extracellular loop regions contribute to proton sensing and channel gating. In particular, the extracellular loop between transmembrane segments S5 and S6 (L5-6) has been implicated in the regulation of channel function (13, 14) (Fig. 2, *A* and *B*). Mutational analysis of human OTOP1 showed that this loop is essential for channel activity and that residue H229 within L5-6 plays a critical role in proton sensing (14). Furthermore, comparative studies of vertebrate OTOP channels indicate that replacing the L5-6 in mouse OTOP2 with that from OTOP3 alters the pH-dependent activation of the OTOP2 channel, supporting a role for this region in regulating OTOP (13). Between human and mouse OTOP3, despite their high overall sequence similarity, L5-6 is among the regions with relatively low sequence conservation (Fig. 2*C*). Since hOTOP3 requires stronger extracellular acidification for activation than mouse OTOP3 (Fig. 1), we tested whether sequence differences within the L5-6 contribute to this species-dependent difference in proton sensitivity.

To examine this possibility, we generated chimeric channels in which the most divergent region within L5-6 was exchanged between human and mouse OTOP3 (Fig. 2*C*). We first replaced the G261-E271 of hOTOP3 with the corresponding region (D242-G252) from mOTOP3 (hOTOP3-mL5-6) and examined proton-activated currents in *Xenopus* oocytes using TEVC. Representative recordings show that this chimera produced slightly larger inward currents at intermediate pH values compared with wild-type (WT) hOTOP3 (Fig. 2*D*). Quantification of normalized currents at −100 mV revealed that hOTOP3-mL5-6 displayed modest but significantly larger currents at pH 4.5 and 3.5 than wild-type hOTOP3 (Fig. 2*E*). Plotting normalized currents as a function of proton concentration showed a slight leftward shift of the activation curve for hOTOP3-mL5-6 relative to WT hOTOP3 (Fig. 2*F*), indicating a modest increase in sensitivity to extracellular acidification.

We next performed the reciprocal experiment by replacing the region D242-G252 of mOTOP3 with the region G261-E271 from hOTOP3 (mOTOP3-hL5-6). In contrast to WT mOTOP3, the chimera exhibited significantly reduced currents at intermediate pH values (Fig. 2, *G* and *H*). Consistent with this reduction, the proton concentration–response relationship of mOTOP3-hL5-6 was markedly shifted toward higher proton concentrations compared with WT mOTOP3 (Fig. 2*I*), indicating that the chimera has reduced sensitivity to proton activation.

Together, these results suggest that L5-6 plays an important role in determining the proton sensitivity of OTOP3 channels and contributes to the species-dependent difference in activation between human and mouse OTOP3.

To gain further insight into the mechanism underlying the species-dependent gating difference, we next performed targeted mutagenesis within the L5-6 region. Our previous study identified H229 as a key determinant of proton sensing in human OTOP1 (14). Because the L5-6 loop of OTOP3 contains no histidine residues, we instead examined whether non-conserved acidic residues, which can be protonated under strong acidic conditions, contribute to the species-dependent gating difference. Sequence alignment revealed that one glutamate residue (E265 in hOTOP3) is conserved in both orthologs, whereas two neighboring positions differ markedly: G261 in hOTOP3 corresponds to D242 in mOTOP3, while E271 in hOTOP3 corresponds to G252 in mOTOP3 (Fig. S1*A*). We therefore generated reciprocal double mutants (hOTOP3-G261D/E271G and mOTOP3-D242G/G252E) to exchange the positions of the glycine and negatively charged residues between the two channels. However, hOTOP3-G261D/E271G exhibited proton sensitivity indistinguishable from WT hOTOP3, whereas the reciprocal mOTOP3 mutant did not reproduce the reduced proton sensitivity observed with the mOTOP3-hL5-6 chimera, despite a modest increase in current amplitude at pH 4.5 (Fig. S1, *B*–*G*). These findings indicate that the species-dependent difference in proton sensing cannot be explained by the positions of these two non-conserved acidic residues alone, but instead likely arises from the combined contributions of multiple residues and/or structural interactions involving the L5-6 loop.

### Strong acidification reveals anion-dependent conductance in human OTOP3

**Figure 3:**
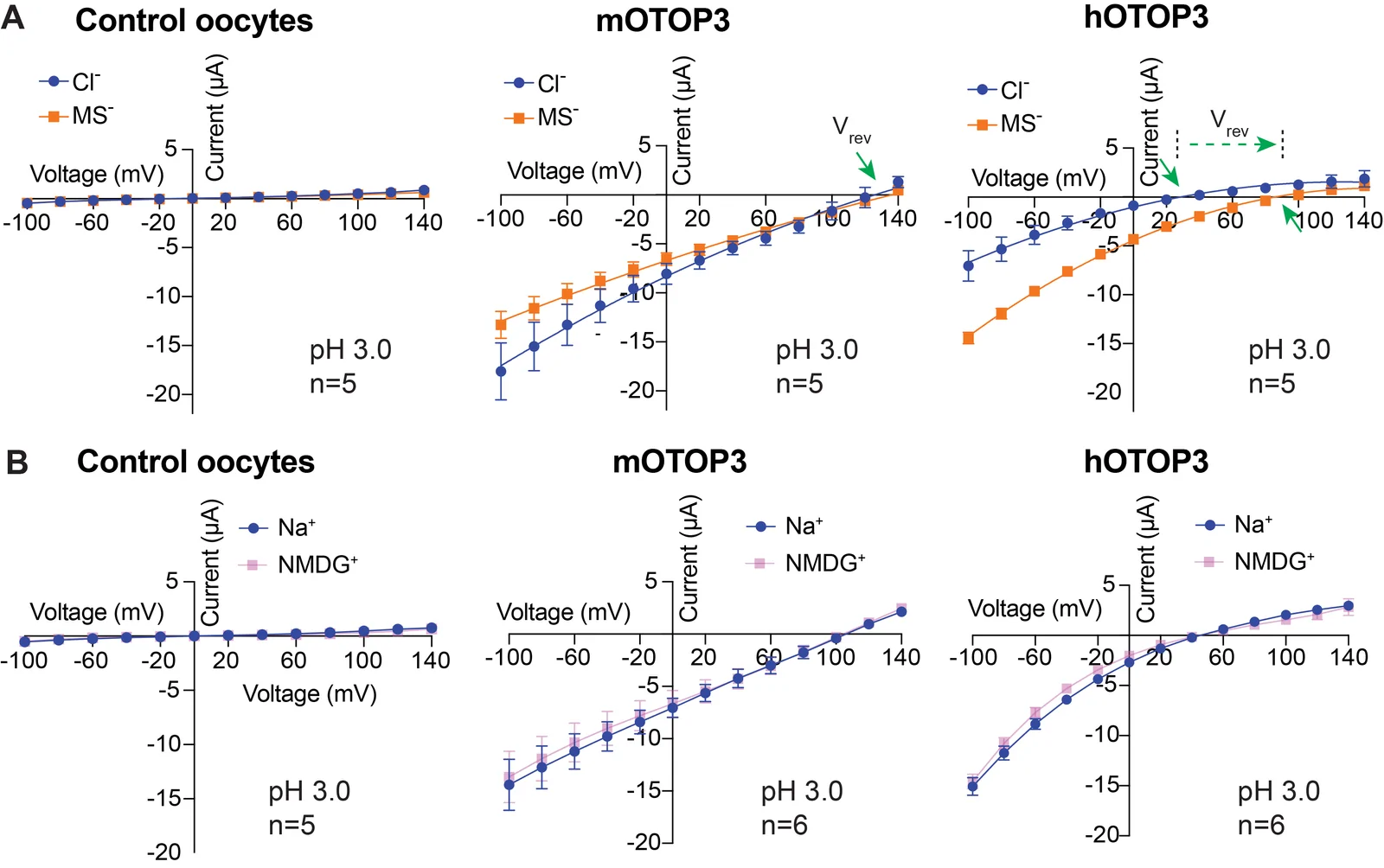
**hOTOP3 exhibits anion-dependent conductance under strongly acidic conditions.***A*, Average I–V curves recorded at pH 3.0 in solutions containing either 100 mM NMDG-Cl or NMDG-methanesulfonate (NMDG-MS). In mOTOP3, currents and V_rev_ were similar in both solutions. In contrast, hOTOP3 showed a pronounced positive shift in V_rev_ in NMDG-MS relative to NMDG-Cl, indicating dependence on the external anion. *B*, average I–V relationships recorded at pH 3.0 in solutions containing either 100 mM NaCl or 100 mM NMDG-Cl. Substitution of Na^+^ for NMDG^+^ did not significantly alter currents or V_rev_ in either channel, indicating minimal contribution from cation permeation. Data are shown as mean ± SD, with the number of oocytes indicated in each panel.

In our recordings, hOTOP3 currents at low pH exhibited a much more negative V_rev_, which may indicate reduced proton selectivity and the presence of an additional non-proton conductance (Fig. 1*B*). To determine whether the outward currents of hOTOP3 in positive voltages reflect influx of the external anions, we compared currents recorded in NMDG-Cl and NMDG-methanesulfonate (NMDG-MS) solutions, in which methanesulfonate is generally considered poorly permeant through anion channels. In mOTOP3, the I-V curves and V_rev_ were similar in both solutions (Fig. 3*A*, middle), consistent with minimal anion permeability. In contrast, the V_rev_ of hOTOP3 shifted markedly in the positive direction (by ∼60 mV) when Cl^−^ was replaced with MS^−^ (Fig. 3*A*, right), indicating that the additional conductance is influenced by external anion (Cl^−^). Meanwhile, substitution of the large cation NMDG^+^ with much smaller Na^+^ in the bath solution did not significantly alter the I–V curves or V_rev_ in either channel (Fig. 3*B*), suggesting that cation permeation does not substantially contribute to the observed outward currents in hOTOP3.

**Figure 4:**
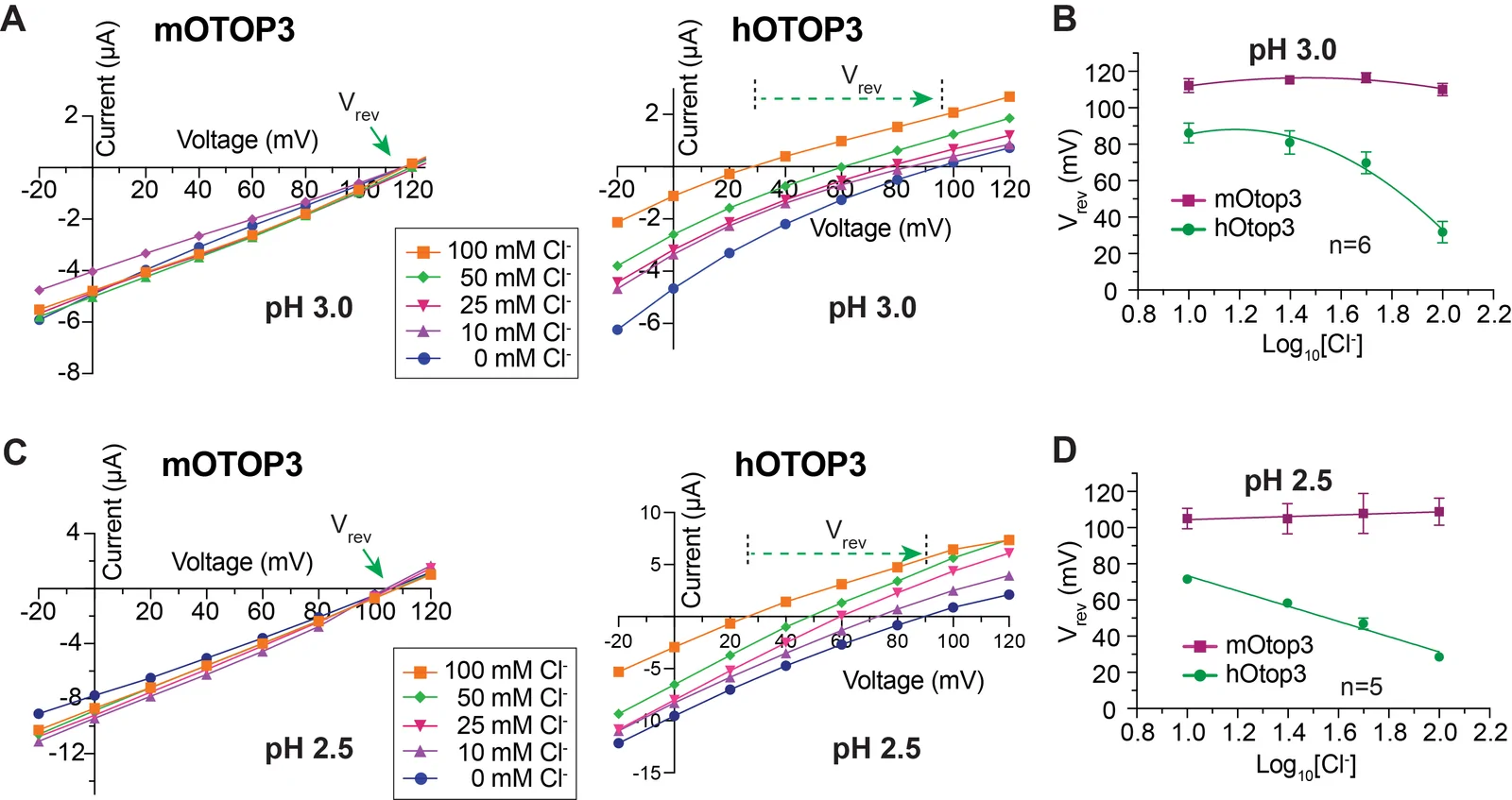
**Reversal potential of hOTOP3 depends on extracellular Cl^−^ concentration.***A, C*, representative I–V relationships of mOTOP3 (*left*) and hOTOP3 (*right*) recorded at pH 3.0 (*A*) and pH 2.5 (*C*) in solutions containing varying extracellular Cl^−^ concentrations (100–0 mM, balanced with methanesulfonate). In mOTOP3, V_rev_ remained largely unchanged across conditions. In contrast, hOTOP3 exhibited a progressive positive shift in V_rev_ with decreasing extracellular Cl^−^ concentration. *B, D*, summary plots of V_rev_ as a function of log_10_[Cl^−^] at pH 3.0 (*B*) and pH 2.5 (*D*). mOTOP3 showed little dependence of V_rev_ on extracellular Cl^−^ concentration, whereas hOTOP3 displayed a strong dependence. The relationship was nonlinear at pH 3.0 but approximately linear at pH 2.5, indicating condition-dependent contributions of Cl^−^ to permeation. Data are presented as mean ± SD; with the number of oocytes indicated in each panel.

We next further tested the dependence of V_rev_ on extracellular chloride concentration. In mOTOP3, decreasing extracellular Cl^−^ concentration had little effect on V_rev_ at either pH 3.0 or 2.5 (Fig. 4, *A* and *C*, left; Fig.4, *B* and *D*), consistent with proton-selective conduction. In contrast, in hOTOP3, V_rev_ gradually shifted to positive as extracellular Cl^−^ concentration decrease at both pH 3.0 and 2.5, consistent with a contribution of Cl^−^ permeability (Fig. 4, *A* and *C*, right; Fig. 4, *B* and *D*). Interestingly, the relationship between V_rev_ and log[Cl^−^] for hOTOP3 was nonlinear at pH 3.0 but approximately linear at pH 2.5 (Fig. 4, *B* and *D*). These results suggest that ion permeation in hOTOP3 is state-dependent, with pH 3.0 likely representing a transitional status near the activation threshold, whereas pH 2.5 favors a more stable mixed-conductance state.

At pH 2.5, decreasing the bath Cl^−^ concentration from 100 mM to 10 mM resulted in a significant rightward shift of V_rev_ from 28.6 ± 2.5 mV to 71.6 ± 1.9 mV in hOTOP3, indicating significant Cl^−^ permeability (Fig. 4, *C*, right and *D*). To quantify the relative contributions of proton and anion permeation, we estimated the apparent permeability ratio *P*_*H*_*/P*_*Cl*_ using the Goldman–Hodgkin–Katz equation based on V_rev_ measured at pH 2.5 under varying extracellular Cl^−^ concentrations (100, 50, 25, 10 mM). Assuming an intracellular Cl^−^ concentration of 30 mM in oocytes (20, 21) and intracellular pH of 7.4, hOTOP3 exhibited average apparent *P*_*H*_*/P*_*Cl*_ value on the order of 10^2^, indicating that the channel remains proton selective but with a substantial contribution from Cl^−^ permeation. In contrast, mOTOP3 exhibited reversal potentials that were largely insensitive to extracellular Cl^−^ concentration (Fig. 4, *C*, left and *D*), consistent with a much higher apparent *P*_*H*_*/P*_*Cl*_ (≫10^3^) and near-exclusive proton selectivity.

Together, these results show that, once activated under strongly acidic conditions, hOTOP3 exhibits a measurable anion contribution to permeation, in contrast to mOTOP3. This behavior indicates that hOTOP3 supports both proton and anion conduction under strongly acidic conditions, with their relative contributions depending on extracellular environment.

### Anion-dependent conductance is specific to human OTOP3 among OTOP family members

**Figure 5:**
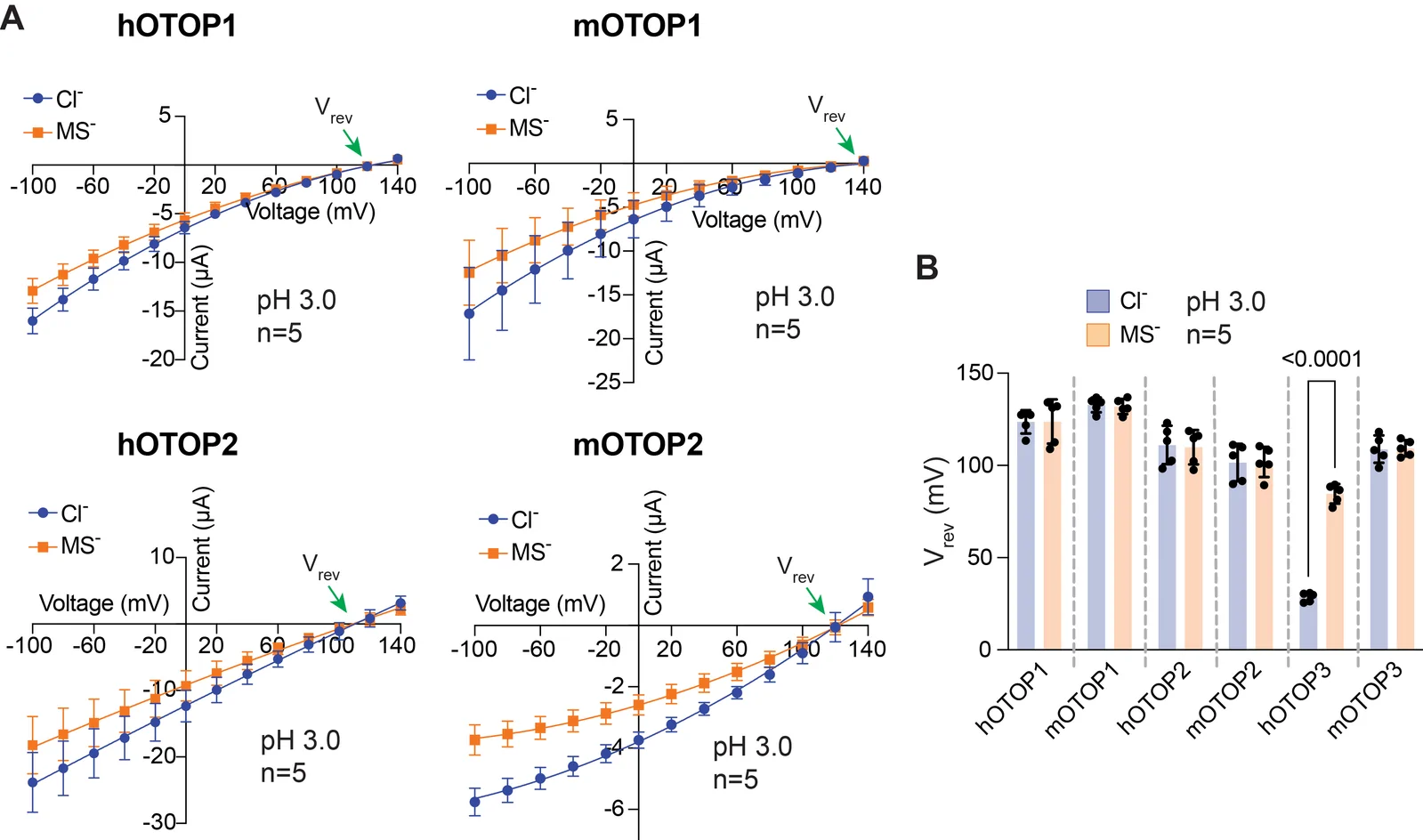
**Anion-dependent conductance is specific to human OTOP3 among OTOP family members.***A*, average I–V relationships recorded from oocytes expressing human and mouse OTOP1 and OTOP2 at pH 3.0 in solutions containing either NMDG-Cl or NMDG-MS. For both OTOP1 and OTOP2 orthologs, I–V relationships and V_rev_ were similar between the two conditions, indicating minimal dependence on the external anion. *B*, summary of V_rev_ values measured in NMDG-Cl and NMDG-MS solutions for OTOP1, OTOP2, and OTOP3 orthologs. No significant differences were observed for OTOP1, OTOP2 and the mouse OTOP3 channels, whereas hOTOP3 showed a significant shift in V_rev_ between conditions, consistent with an anion-dependent contribution to permeation. Statistical significance was assessed with Student’s *t* test. Statistically significant comparisons are indicated by their corresponding *p* values; comparisons without labels were not statistically significant. Data are presented as mean ± SD; individual data points represent independent oocytes, with n = 5. Data for hOTOP3 and mOTOP3 were extracted from Figure 3*A*.

To determine whether anion-dependent conductance under strongly acidic condition is a unique feature of hOTOP3 channel, we examined human and mouse OTOP1 and OTOP2 under the same conditions used for OTOP3. At pH 3.0, I-V curves recorded in NMDG-Cl and NMDG-MS solutions were largely indistinguishable for both hOTOP1 and mOTOP1, as well as hOTOP2 and mOTOP2 (Fig. 5*A*). In all cases, reversal potentials were similar between the two anion conditions, indicating minimal contribution of anion conductance.

Quantification of V_rev_ confirmed that substitution of Cl^−^ with methanesulfonate did not produce significant changes for OTOP1 and OTOP2 orthologs and mOTOP3 (Fig. 5*B*). In contrast, hOTOP3 exhibited a marked shift in V_rev_ between the two conditions, consistent with a substantial contribution of anion-dependent conductance (Fig. 5B).

These results indicate that anion-dependent conductance at low pH is not a general property of OTOP channels but instead appears to be a distinct feature of hOTOP3.

### Mutations of a conserved N-pore residue coordinately alter proton and anion conduction in hOTOP3

**Figure 6:**
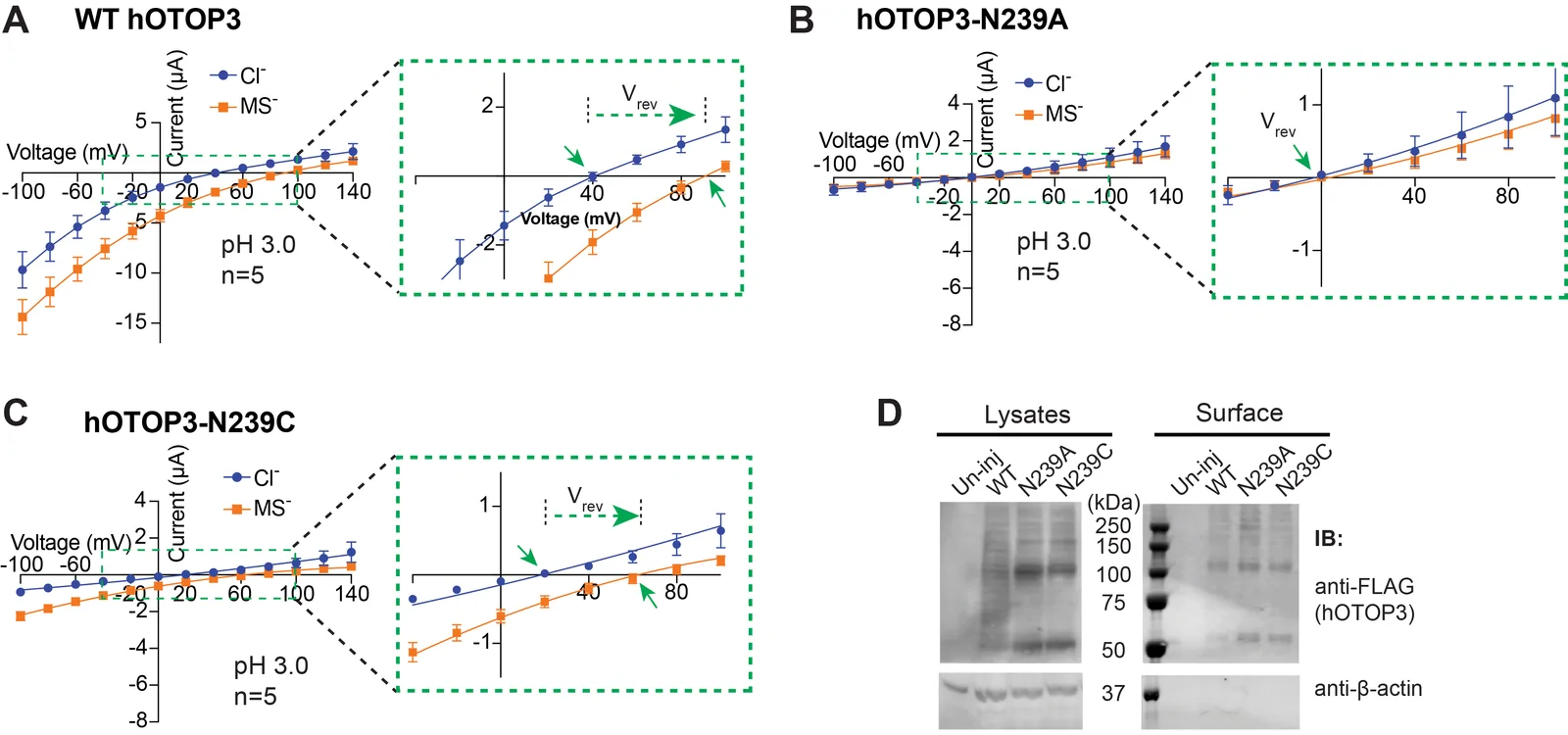
**A conserved N-domain pore residue coordinately regulates proton and anion conduction in hOTOP3.***A, B, and C*, average I–V relationships of WT hOTOP3 (*A*) and mutants N239A (*B*) and N239C (*C*) recorded at pH 3.0 in NMDG-Cl and NMDG-MS. N239A exhibited minimal currents with no difference between conditions, whereas N239C retained currents and showed clear differences between Cl^−^ and MS^−^, indicating partial preservation of both proton and anion conduction. Insets show expanded views near the V_rev_. Data are presented as mean ± SD; n values are indicated in each panel. *D*, surface biotinylation followed by Western blot analysis shows comparable overall and surface expression levels among WT hOTOP3 and the two N293 mutants. OTOP3 samples displayed multiple bands, likely reflecting glycosylation, which were absent in control oocytes. Although the predicted molecular weights of the monomer and dimer are ∼66.3 kDa and ∼132.6 kDa, respectively, the major bands observed were smaller (∼55 kDa and ∼110 kDa), possibly due to cleavage or partial degradation.

To investigate whether proton and anion conduction are mechanistically linked in hOTOP3, we targeted a conserved residue N239 within the conserved glutamine-asparagine-tyrosine constriction triad in the N-pore, which has been previously shown to be essential for proton conduction (11, 12). At pH 3.0, the N239 A mutant exhibited minimal currents comparable to WT hOTOP3, and no discernible differences were observed in currents recorded in NMDG-Cl and NMDG-MS solutions (Fig. 6, *A* and *B*), indicating that both proton and anion conductance were abolished. In contrast, the N239C mutant retained small but measurable inward currents and displayed clear differences V_rev_ between Cl^−^ and MS^−^ conditions (Fig. 6*C*), consistent with partial preservation of both proton and anion conductance.

To determine whether the loss of current in N239A resulted from impaired surface expression, we performed surface biotinylation experiments. Both N239A and N239C were detected at the plasma membrane at levels comparable to WT hOTOP3 (Fig. 6*D*), indicating that the marked reduction in channel activity is unlikely to result from defective membrane trafficking.

Together, these results demonstrate that mutations at N239 coordinately affect both proton and anion conduction while preserving surface expression. These findings support the idea that proton and anion conduction are mechanistically coupled and depend on a common functional element involving the N-pore. However, they do not establish whether protons and anions permeate through the same physical pathway, which will require future structural and mechanistic studies.

## Discussion

Previous studies have shown that OTOP channels exhibit distinct pH-dependent gating properties across species and family members. In mouse and human OTOP1 expressed in heterologous systems, extracellular acidification robustly activates proton currents with thresholds near pH ≤ 6.0, with current amplitudes increasing progressively at lower pH (4, 13, 14). Notably, OTOP1 from multiple species have also been shown to be activated by extracellular alkalinization, supporting bidirectional proton flux and indicating the presence of distinct gating mechanisms for acid and base stimuli (13, 22). Recordings of *Xenopus tropicalis* OTOP3 indicate a similar activation threshold near pH ≤ 6.0 (12). However, in contrast, mouse OTOP3 exhibits a more restricted activation profile, requiring stronger acidification (typically pH < 5.5) to elicit measurable currents, with steep increases in activity at lower pH (4, 13). In contrast to OTOP1 and OTOP3, mouse OTOP2 displays broader activity across pH conditions, remaining active at near-neutral pH and exhibiting outward currents under alkaline conditions, suggesting a more constitutively active or weakly gated channel (4, 13, 18, 19).

Together, these findings indicate that OTOP channels are differentially tuned to specific physiological pH environments across species, with OTOP1 supporting both acid- and alkali-dependent activation, whereas OTOP3 orthologs consistently require stronger acidification for activation. Our results further extend this framework by demonstrating that human OTOP3 requires even stronger acidification for activation, with little or no activity observed at pH ≥ 4.5 and robust currents only at pH ≤ 3.5 (Fig. 1). This distinctive gating profile suggests that human OTOP3 is tuned to operate under more extreme acidic conditions than previously characterized OTOP3 orthologs and may therefore fulfill a specialized physiological role in highly acidic microenvironments.

Compared to mOTOP3, hOTOP3 requires substantially lower pH to open (Fig. 1). Domain-swapping experiments between the two implicate the extracellular L5-6 as an important determinant of this gating difference (Fig. 2), extending previous structural and functional work suggesting that extracellular elements contribute to proton-dependent activation (13, 14). These results indicate that relatively small sequence variations can tune the energetic barrier for channel opening and shift the functional operating range of different OTOP family members.

All studies reported to date have consistently shown that OTOP channels exhibit high proton selectivity, supporting the prevailing view that these channels function as dedicated proton conductors (4, 12, 22). This strict selectivity has been considered a defining feature of the OTOP family, reflecting structural adaptations that favor proton permeation while excluding other ions. However, our findings reveal that human OTOP3 displays measurable Cl^−^ permeability under strongly acidic conditions (Fig. 3), indicating that ion selectivity in this channel is not absolute but can depend on its activation state. Notably, this anion contribution becomes more pronounced and stabilized as the pH decreases from 3.0 to 2.5 (Fig. 4), suggesting that extreme protonation may alter the electrostatic environment and/or conformational state of the channel, thereby changing its ion permeation properties.

Consistent with this idea, mutations of the conserved N-pore residue N239 coordinately altered both proton and anion conduction while preserving surface expression, indicating that the two processes are mechanistically linked. However, these findings do not establish whether protons and anions permeate through the same physical pathway. Instead, they suggest that proton and anion conduction depend on a common functional role of the N-pore, either through a shared permeation pathway or through closely coupled structural mechanisms.

This observation is consistent with evidence from other ion channel systems indicating that proton selectivity can be modulated by channel state and environmental conditions. For example, mutation of a key negatively charged residue in the proton pathway of the voltage-gated proton channel Hv1 has been shown to convert the channel from proton-selective to anion or cation-permeable, demonstrating that subtle changes in pore electrostatics can dramatically alter proton selectivity (23, 24). In addition, the lysosomal channel TMEM175 was recently reported to exhibit proton-selective conductance under acidic conditions while retaining permeability to other ions (25), indicating that proton conductance can be dynamically tuned by environmental pH. Together, these studies highlight that proton selectivity is not an immutable property but rather an emergent feature arising from channel structure, protonation state, and the surrounding electrostatic landscape. In this context, the condition-dependent mixed proton–anion conductance observed in hOTOP3 suggests that some OTOP channels may also possess greater functional flexibility than previously appreciated, with their proton selectivity modulated by environments.

The functional differences observed among OTOP family members, together with their distinct expression patterns, suggest physiological specialization across tissues. Transcriptomic studies have shown that OTOP3 is expressed in the stomach, epidermis, retina, and small intestine (4, 26), although its precise cellular localization and physiological function remain unknown. Among these tissues, the stomach is unique in containing regions exposed to highly acidic luminal contents, where pH can fall below 3. The unusually strong acid requirement of hOTOP3 therefore may represent an adaptation that restricts channel activation to conditions of extreme acid exposure, rather than the more moderate acidification sufficient to activate other OTOP channels. The mixed conductance observed in this study further raises the possibility that hOTOP3 contributes to coupled proton–anion flux under such conditions, which could help maintain electrochemical balance during proton influx. Notably, we also observed reduced inactivation of hOTOP3 compared with mOTOP3 at pH 2.5 (Fig. 1*A*). One possible explanation is that the anion permeability of hOTOP3 helps dissipate intracellular positive charge accumulation during proton influx, thereby stabilizing channel activity. This idea is consistent with previous findings in *X. tropicalis* OTOP3, where proton-induced inactivation has been attributed, at least in part, to intracellular charge buildup (12). Although this hypothesis remains speculative, it raises the possibility that the unique permeation properties of hOTOP3 help sustain channel activity during prolonged exposure to strong acid. Together, these observations support a model in which OTOP channels are functionally specialized rather than redundant, with hOTOP3 adapted for operation in highly acidic physiological environments.

Several limitations should be noted. All measurements were performed in a heterologous expression system (*Xenopus* oocytes), and the extent to which mixed conductance occurs in native tissues remains unknown. In addition, although our data demonstrate that proton and anion conduction are mechanistically coupled, they do not distinguish whether the two ions permeate through the same physical pathway or through distinct but closely coupled pathways. Resolving this question will require future structural, computational, and functional studies.

In summary, our findings expand the functional diversity of the OTOP channel family by identifying hOTOP3 as a channel with a higher activation threshold and condition-dependent mixed proton-anion conductance. These results reveal a previously unrecognized flexibility in ion selectivity within the OTOP family and suggest that hOTOP3 may be adapted to function in strongly acidic microenvironments, where coupled proton and anion flux may help maintain electrochemical balance and sustain channel activity.

## Experimental procedures

### cDNA constructs and cloning

The cDNAs encoding human OTOP1 (NCBI accession #BC130430.1), mouse OTOP1 (NCBI accession #NM_172709.3), human OTOP2 (NCBI accession #NM_178160.3), mouse OTOP2 (NCBI accession #NM_001362400.1), mouse OTOP3 (NCBI accession #NM_001347647.2) and human OTOP3 (NCBI accession #NM_001272005.2) were cloned separately into a modified pGEMHE vector for *in vitro* transcription. All mutations were introduced by PCR-based mutagenesis and verified by DNA Sanger sequencing. All OTOP channels used in this study contain an N-terminal FLAG tag.

### Electrophysiology: Oocyte injection and solutions

#### Oocytes and solutions

Stage V–VI oocytes were harvested and prepared from adult female *X. laevis* following standard procedures (27). cDNA constructs were linearized and used for *in vitro* synthesis of RNA with T7 RNA polymerase. RNA (50 ng/oocyte) was injected into oocytes with a Drummond Scientific Nanoject III injector. Injected oocytes were incubated at 18 °C for 3 to 4 days before electrophysiology recording.

The standard bath solution for TEVC recordings contained 100 mM NMDG-Cl, 1 mM MgCl_2_, and 2 mM HEPES, adjusted to pH 7.5. For acidic solutions, 5 mM MES replaced HEPES in the pH 6.5 and 5.5 solutions, whereas 5 mM Homo-PIPES was used for the pH 4.5 solution. The pH 3.5 solution contained 10 mM trisodium citrate and 5 mM citric acid and was adjusted to pH 3.5 with HCl. Solutions at pH 3.0 and 2.5 were buffered with 20 mM glycine and adjusted to the desired pH with HCl or methanesulfonic acid. The final pH of all solutions was verified immediately before use.

To assess chloride permeability, the standard neutral-pH solution contained 100 mM NMDG-methanesulfonate (NMDG-MS), 1 mM MgSO_4_, and 2 mM HEPES, adjusted to pH 7.5. Acidic solutions contained either 100 mM NMDG-MS with 1 mM MgSO_4_ and 20 mM glycine (adjusted to pH 3.0 with methanesulfonic acid) or 100 mM NMDG-Cl with 1 mM MgCl_2_ and 20 mM glycine (adjusted to pH 3.0 with HCl).

Solutions with extracellular Cl^−^ concentrations of 100, 50, 25, 10, or 0 mM were prepared from 100 mM NMDG base by adding the appropriate amounts of HCl. Methanesulfonic acid was then added to provide the replacement anion and to adjust the final pH to 3.0. All solutions contained 20 mM glycine.

### Electrophysiology: Voltage clamp and protocols

Whole-cell currents from *X. laevis* oocytes were recorded using the two-electrode voltage clamp (TEVC) technique with an OC-725C Oocyte Clamp amplifier (Warner Instruments), a Digidata 1440A digitizer (Molecular Devices), and the pClamp 10 software (Molecular Devices).

Voltage protocols, including gap-free and voltage steps were applied during TEVC recording. For gap-free recording, oocytes were clamped at −60 mV in the standard bath and low-pH bath solutions were applied for roughly 20 s to invoke channel currents at different pHs. To obtain the current–voltage (I–V) relationship at a certain pH, oocytes were clamped at +50 mV and 40 ms voltage steps from +100 to −100 mV were applied in 10 mV increments and the data were used to extract the I–V curves. The positive holding potential and voltage steps from positive to negative were employed to minimize proton efflux at positive voltages. In contrast, holding oocytes at negative potentials and applying negative voltage steps first promotes intracellular proton accumulation, which can artifactually enhance outward proton currents at positive voltages and affect the V_rev_ measurement.

To obtain accurate reversal potentials in chloride and methanesulfonate comparison, including conditions with variable chloride concentrations, a tail current protocol was employed. Oocytes were held at +40 mV for 60 ms, followed by voltage steps from +140 to −100 mV in 20 mV increments for 20 ms. Tail currents were used to generate I–V relationships.

### Electrophysiology: Permeability ratio calculation

Apparent permeability ratios for H^+^ and Cl^−^ were estimated from reversal potentials using the Goldman–Hodgkin–Katz voltage equation, assuming permeability to H^+^ and Cl^−^:

$$V_{rev}=\frac{RT}{F}\ln\left(\frac{P_{H}{\left[H^{+}\right]}_{o}\;+\;P_{Cl}{\left[Cl^{-}\right]}_{i}}{P_{H}{\left[H^{+}\right]}_{i}\;+\;P_{Cl}{\left[Cl^{-}\right]}_{o}}\right)$$

where *R* is the gas constant, *T* is absolute temperature, and *F* is Faraday’s constant. All ion concentrations were expressed in mM. Intracellular pH was assumed to be 7.4, and intracellular Cl^−^ concentration was assumed to be 30 mM for *Xenopus* oocytes (20, 21). Apparent *P*_*H*_*/P*_*Cl*_ values were calculated from measured reversal potentials obtained at different extracellular Cl^−^ concentrations. Because intracellular ion concentrations were not directly measured, the calculated permeability ratios represent apparent estimates.

### Oocyte surface biotinylation, SDS-PAGE, and Western blot

Proteins expressed on *Xenopus* oocyte plasma membrane were detected with the Pierce Cell Surface Biotinylation and Isolation kit following a modified protocol described previously (28). Briefly, 2 to 3 days after RNA injection, oocytes (30 per group) were collected and washed twice with cold OR2 solution (82.4 mM NaCl, 2.5 mM KCl, 1 mM MgCl_2_, 10 mM HEPES, pH 7.6). Oocytes were then incubated with 0.25 mg/ml sulfo-NHS-SS-biotin in ice-cold OR2 at room temperature for 1 h. The reaction was then quenched, and oocytes were washed, following the manufacturer’s protocol. Oocytes were then kept in a lysis solution containing 1× PBS, 5 mM EDTA, 1% Nonidet P-40 (NP-40), and 1/50 (v/v) Protease Inhibitor Cocktail (Sigma). They were first homogenized and then incubated by rotating at 4 °C for 1 h. Lysates were centrifuged for 30 min at 10,000*g*, and supernatants were collected. Lysates were then mixed with NeutrAvidin Agarose at 4 °C overnight. After beads were washed, proteins were eluted with 50 mM DTT at 37 °C for 30 min.

Eluted samples were analyzed by SDS–PAGE and Western blot. Oocyte lysate and surface biotinylation samples were run on 4 to 12% Bolt Bis-Tris Plus gels (Thermo Fisher Scientific) and transferred to a PVDF membrane. Mouse monoclonal anti-FLAG primary antibody (MilliporeSigma), mouse monoclonal anti-β-actin antibody (GenScript), and the LI-COR IRDye 680RD goat anti-mouse secondary antibody (LICORbio) were used for blotting. Blot signals were visualized with LI-COR Odyssey.

### Statistical analysis

Statistical analyses were performed using GraphPad Prism (GraphPad Software). For proton sensing experiments, currents recorded from the same cells across multiple pH conditions were analyzed using two-way ANOVA. Sidak’s multiple-comparisons test was used for *post hoc* comparisons between human and mouse or WT and mutant at each pH. For V_rev_ shift analysis in Figure 5, statistical significance was determined by Student’s *t* test. Data are presented as mean ± SD.

### Animal use

*X. laevis* oocytes were obtained from female frogs. All animal care and experimental procedures were conducted in accordance with protocols approved by the Institutional Animal Care and Use Committee (IACUC) at St John’s University.

## Data availability

All data are contained within the manuscript.

## Supporting information

This article contains supporting information.

## Conflict of interest

The authors declare that they have no conflicts of interest with the contents of this article.
