# Supplementary material for: Human OTOP3 requires strong acidification for activation and exhibits mixed proton-anion permeation
Source: J Biol Chem. 2026 Aug 14;302(10):113454. doi: 10.1016/j.jbc.2026.113454 (PMC13579931; doi:10.1016/j.jbc.2026.113454)
Supplement: Supplementary file 1 [file mmc1.docx]

**Human OTOP3 requires strong acidification for activation and exhibits mixed proton-anion conduction**

Tiago D. C. Morais, Fatemeh M. Badizi, Itai Itzhak, Moushumi A. Mou, Justin Chan, Anonnya Debi, Zhifei Wang, Yong Yu*

Department of Biological Sciences, St. John’s University, Queens, New York, 11439, USA

*** To whom correspondence should be addressed:**

Yong Yu

Department of Biological Sciences, St. John’s University, St. Albert Hall 210, 8000 Utopia Parkway, Queens, NY 11439, USA

Phone: 1 (718)-990-1654;	Email: [yuy2@stjohns.edu](mailto:yuy2@stjohns.edu)

**Material included: Figure S1.**

**Figure S1. Reciprocal substitutions of two non-conserved residues within the L5-6 loop do not reproduce the species-dependent gating phenotype.**
**(A)** Sequence alignment of the L5-6 regions of human and mouse OTOP3. Arrows indicate the two non-conserved positions selected for reciprocal substitution. **(B, E)** Representative TEVC current traces recorded from WT and mutant channels in response to extracellular acidification. **(C, F)** Summary of normalized current amplitudes at -100 mV measured at different extracellular pH values. Data are presented as mean ± SD; sample sizes are indicated in parentheses. Currents were normalized to the current recorded at pH 2.5 for each oocyte. Statistical significance was assessed using two-way ANOVA with Sidak’s multiple-comparisons test. Statistically significant comparisons are indicated by their corresponding P values; comparisons without labels were not statistically significant. **(D, G)** Proton concentration–response relationships derived from normalized currents at −100 mV for the indicated channels (mean ± SD). Reciprocal exchange of G261/D242 and E271/G252 did not reproduce the marked changes in proton sensitivity observed with the L5–6 chimeras, indicating that these residues alone are insufficient to account for the species-dependent gating differences.
